# Immortalized Schwann cell lines as useful tools for pathogenesis-based therapeutic approaches to diabetic peripheral neuropathy

**DOI:** 10.3389/fendo.2024.1531209

**Published:** 2025-01-21

**Authors:** Kazunori Sango, Hideji Yako, Naoko Niimi, Shizuka Takaku

**Affiliations:** ^1^ Diabetic Neuropathy Project, Tokyo Metropolitan Institute of Medical Science, Tokyo, Japan; ^2^ Laboratory of Molecular Neuroscience and Neurology, Tokyo University of Pharmacy and Life Sciences, Tokyo, Japan

**Keywords:** immortalized Schwann cells, diabetic peripheral neuropathy, polyol pathway, glycation, oxidative stress, autophagic and proteostatic disturbances

## Abstract

Growing evidence suggests that hyperglycemia-related abnormalities in Schwann cells play a pivotal role in the development and progression of diabetic peripheral neuropathy (DPN). Several immortalized Schwann cell lines have been established in our laboratory and utilized for the study of DPN; IMS32 from normal ICR mice, 1970C3 from normal C57BL/6 mice, IWARS1 and IKARS1 from wild-type and aldose reductase-deficient C57BL/6 mice, and IFRS1 from normal Fischer 344 rats. These cell lines retain biological features of Schwann cells and display high proliferative activities that enable us to perform molecular and biochemical analyses. In addition, these cells have exhibited metabolic alterations under exposure to diabetes-associated conditions, such as hyperglycemia, dyslipidemia, glycative and oxidative stress load. Herein, recent studies with these cell lines regarding the pathogenic factors of DPN (augmentation of the polyol and other collateral glycolysis pathways, glycative and oxidative stress-induced cell injury, autophagic and proteostatic disturbances, etc.) and therapeutic strategies targeting these factors are introduced.

## 1 Introduction

As glial cells in the peripheral nervous system (PNS), Schwann cells are responsible for providing trophic support for the growth and maintenance of neurons and wrapping their axons in either a myelinating or an unmyelinating form. Following axonal injury, Schwann cells dedifferentiate into a ‘repair’ phenotype, contributing to axonal regeneration and functional recovery. They proliferate, migrate to the lesion site, eliminate axon debris alongside macrophages, promote the elongation of new axons, and finally remyelinate them (1, 2).

**Figure 1:**
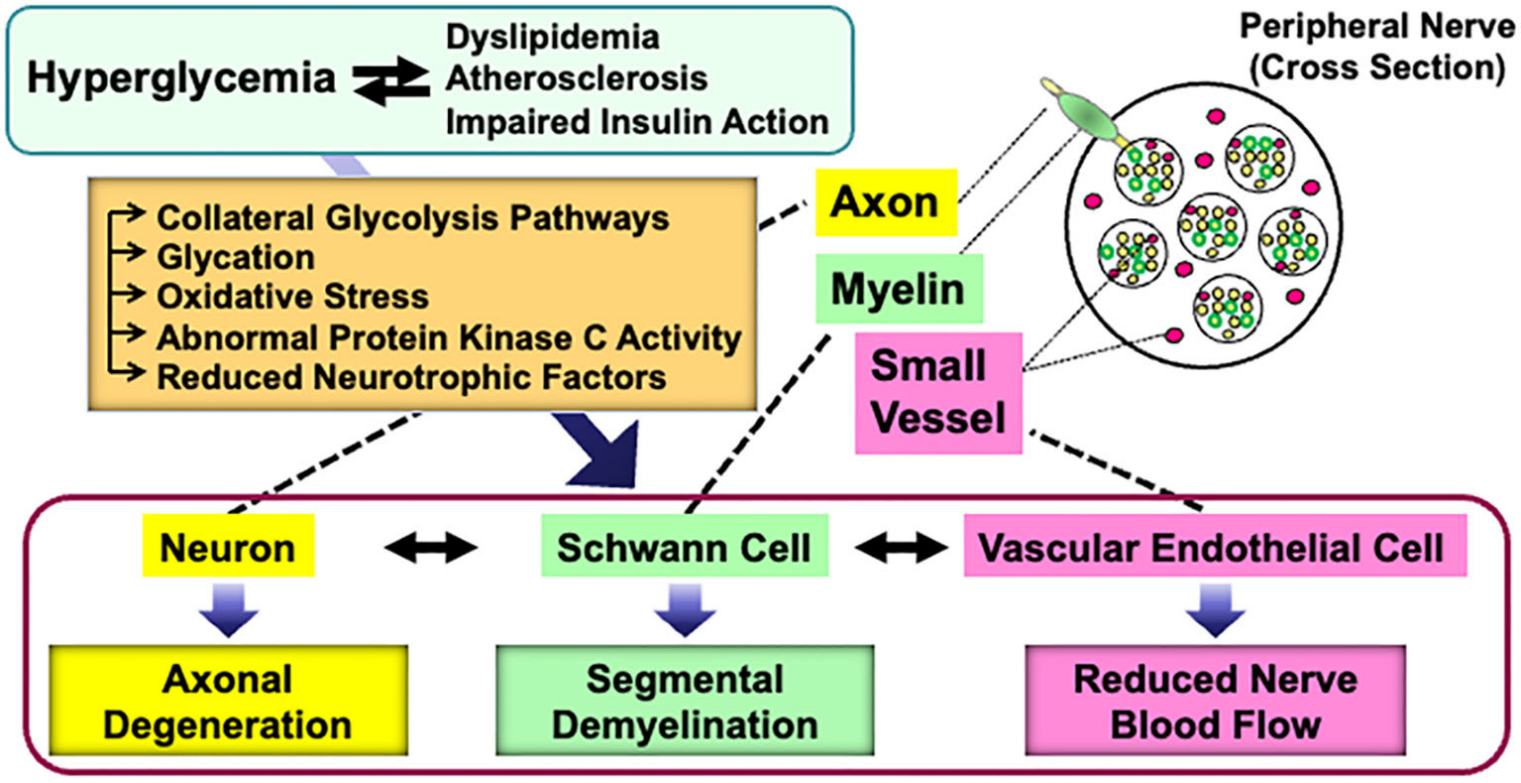
Current understanding of the pathogenesis of DPN. Details are explained in the text.

Abnormalities of Schwann cells and/or their crosstalk with neurons and other non-neuronal cells in the PNS can lead to various types of peripheral neuropathies. *Diabetes Mellitus* is one of the major causes of neuropathies, and diabetic peripheral neuropathy (DPN) is the most frequent and early-onset complication of type 1 and type 2 diabetes (3, 4). DPN is characterized by progressive, nerve-length-dependent loss of peripheral nerve fibers, leading to impaired sensory and autonomic function, pain, numbness, and eventually, complete loss of sensation. Although its pathogenesis remains largely unclear, hyperglycemic insults, along with dyslipidemia, atherosclerosis, and impaired insulin action (in the case of type 2 diabetes), cause a range of metabolic abnormalities in neurons, Schwann cells, and vascular endothelial cells. These changes result in axonal degeneration, demyelination, and reduced nerve blood flow, respectively (5). Furthermore, the abnormalities in each cell type can trigger dysfunction in others (6) (**Figure 1**).

Cultured Schwann cells are valuable tools to explore the pathogenesis of DPN. Methods for primary culture of Schwann cells from both normal and diabetic animals have been established and widely used in DPN research (7–9). However, the primary cultures have several disadvantages, including time-consuming process, ethical concerns related to animal sacrifice, and an insufficient cell population for molecular and biochemical analyses. To address these issues, several Schwann cell lines derived from schwannoma tissues and long-term primary cultures have been developed as novel *in vitro* models for DPN (10, 11). Notably, spontaneously immortalized Schwann cell lines exhibit high proliferative activity while largely retaining the distinct phenotypes of Schwann cells, making them ideal for studying pathogenic mechanisms (12). Although the detailed mechanisms underlying cell immortalization remain unclear, this phenomenon is thought to represent an early step in the progression toward cellular transformation (13). Unlike primary cultured cells, which have a limited lifespan, immortalized cells can be passaged indefinitely and are not contact-inhibited when cultures reach confluence. However, most immortalized Schwann cells exhibit mitogenic responses to growth factors (e.g., transforming growth factor-β, platelet-derived growth factor, acidic and basic fibroblast growth factors, etc.) and secrete various neurotrophic factors in a manner similar to primary cultured Schwann cells (13–18). Additionally, both primary cultured and immortalized Schwann cells undergo differentiation (e.g., increased expression of myelin proteins and the Krox-20 transcription factor) and dedifferentiation (e.g., increased expression of SOX2 and c-Jun) in responses to genetic manipulation, chemical stimuli, and co-culture with neurons (19–23). These findings suggest that immortalized Schwann cells retain several characteristic features of primary cultured Schwann cells. We previously reported on immortalized Schwann cell lines established in our laboratory, such as IMS32 from ICR mice (15) and IFRS1 from Fischer 344 rats (17), which have proven to be useful tools in DPN research (24). Since then, numerous studies using these cell lines have been conducted by us and other researchers. Additionally, new Schwann cell lines have been established from both normal and aldose reductase (AR)-deficient C57BL/6 mice, including 1970C3 (18), IWARS1 (25, 26), and IKARS1 (18, 25) (**Table 1**). In this article, we briefly summarize the findings obtained from these cell lines, which are expected to contribute to the development of pathogenesis-based therapies for DPN.

**Table 1:** Spontaneously immortalized rodent Schwann cell lines described in this article.

Cell Line	Origin (Species)	Strain	Characteristics	References
IMS32	Normal mice	ICR	High proliferative activity Enhanced polyol pathway activity under high glucose conditions	Watabe et al., J. Neurosci. Res. (1995) (15) Sango et al., J. Neurochem. (2006) (27)
1970C3	Normal mice	C57BL/6	Enhanced polyol pathway activity under high glucose conditions	Niimi et al., J. Neurochem. (2018) (18)
IWARS1	Normal (AR-wild type) mice	C57BL/6	Enhanced polyol pathway activity under high glucose conditions	Suzuki et al., iScience (2023) (26) Yamaguchi et al., J. Biol. Chem. (2024) (28)
IKARS1	AR-deficient mice	C57BL/6	Inactive polyol pathway	Niimi et al., J. Neurochem. (2018) (18) Yamaguchi et al., J. Biol. Chem. (2024) (28)
IFRS1	Normal rats	Fischer344	Myelination in co-culture with primary cultured and lined neurons	Sango et al., J. Neurosci. Res. (2011) (17)

## 2 IMS32 cells

IMS32 cells, one of the best-characterized immortalized Schwann cells, spontaneously arose from long-term primary cultures of adult ICR mouse dorsal root ganglia (DRG) and peripheral nerves. During the purification process, complement and anti-Thy1.2 antibody were used to eliminate fibroblasts from the culture (15). IMS32 cells display distinct Schwann cell phenotypes, including spindle-shaped morphology with immunoreactivity for glial cell markers, and synthesis and secretion of neurotrophic factors; however, there have been no evidence that the cells could myelinate neurites in co-culture with neurons. The high proliferative activity of IMS32 cells might disturb continuous and stable neuron-Schwann cell interactions. Despite this problem, IMS32 cells are recognized as a useful tool to study the action mechanisms of axonal regeneration-promoting factors (29–33), as well as the pathogenesis of neurodegenerative disorders (34–36) and glial cell-associated cancer metastasis (37).

As previously reported (24), IMS32 cells have been utilized for exploring the pathogenesis of DPN, such as polyol pathway hyperactivity (27), glycation (38), and reduced NGF secretion (39). Herein, several important studies published after 2011 will be introduced.

### 2.1 Schwann cell dedifferentiation

As stated in Introduction [1], Schwann cells undergo dedifferentiation following peripheral nerve injury as a prerequisite for successful axonal regeneration. In contrast, Schwann cell dedifferentiation is suggested to play a pathological role in peripheral neuropathies, including hereditary neuropathy (40), autoimmune neuritis (41), chemotherapy-induced neuropathy (42), and DPN. Neureguin-1/ErbB2 signaling, which promotes Schwann cell differentiation, was impaired in the peripheral nerves of diabetic mice (43). Additionally, hyperglycemic insults induced dedifferentiation of primary cultured and IMS32 Schwann cells, evidenced by reduced expression of myelin protein zero (MPZ) and enhanced expression of p75, a low-affinity neurotrophin receptor and marker of immature Schwann cells (44). Schwann cell dedifferentiation under diabetic conditions may lead to Schwann cell death and demyelination; however, the main pathology of DPN is believed to be axonal degeneration rather than demyelination, with the latter being evident in DPN patients only at advanced stages (45). Since the interaction between axons and Schwann cells is essential for maintaining peripheral nerve function, discordance arising from Schwann cell dedifferentiation might affect both myelinated and unmyelinated fibers in DPN. Conversely, the induction of Schwann cell differentiation could be a potential therapy for DPN (46). Transplantation of human mobilized mononuclear cells (hMNC) restored the amplitude of compound muscle action potentials and MPZ expression in the sciatic nerves of diabetic nude rats. Furthermore, co-cultured hMNC induced MPZ expression, along with morphologic maturation of IMS32 cells (47). These findings suggest that the ameliorating effects of hMNC on DPN can, at least partly, be attributed to Schwann cell differentiation.

### 2.2 Glucosamine toxicity

**Figure 2:**
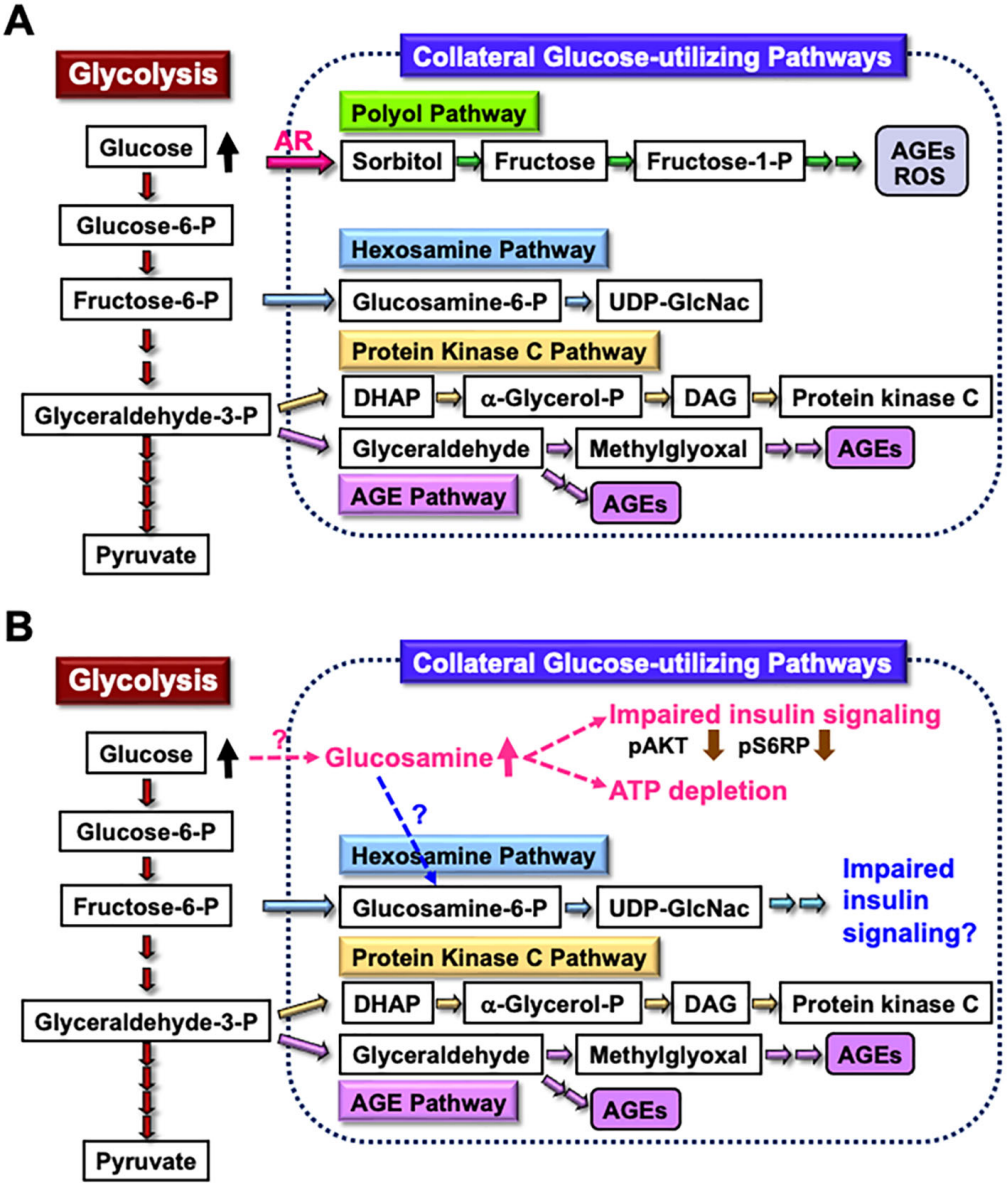
Glucosamine as a novel pathogenic factor in DPN. **(A)** Schematic representation of glycolysis and collateral glucose-utilizing pathways in Schwann cells. **(B)** Regardless of the presence or absence of polyol pathways, increased glucosamine uptake into Schwann cells under diabetic conditions, through a specific mechanism, may impair the insulin signaling pathway and reduce ATP synthesis. Additionally, glucosamine can be metabolized into glucosamine-6-phosphate (Glucosamine-6-P), a key intermediate in the hexosamine pathway, which may further contribute to impaired insulin signaling. These abnormalities may play a significant role in the development of DPN. Abbreviations: Glucose-6-P, glucose-6-phosphate; Fructose-6-P, fructose-6-phosphate; Glyceraldehyde-3-P, glyceraldehyde-3-phosphate; AR, aldose reductase; AGEs, advanced glycation endproducts; ROS, reactive oxygen species; UDP-GlcNAc, uridine diphosphate N-acetyl-D-glucosamine; DHAP, dihydroxyacetone phosphate; α-Glycerol-P, α-glycerol-phosphate; DAG, diacylglycerol; pS6RP, phospho-S6 ribosomal protein.

The enhanced AR activity and polyol pathway flux in the PNS under hyperglycemic conditions are believed to play a major role in the development of DPN (25). When AR-deficient mice were rendered diabetic through streptozotocin (STZ) treatment, they did not exhibit overt neurological symptoms 12 weeks after the onset of diabetes (48). However, reduced nerve conduction velocities were observed in both wild-type and AR-deficient diabetic mice 16 weeks after STZ injection (49). These findings suggest that other pathways, either downstream of or independent of the polyol pathway, contribute to the development of DPN in prolonged diabetes. Metabolomics analysis revealed elevated glucosamine levels in the sciatic nerves of both wild-type and AR-deficient mice exposed to 12 weeks of diabetes. Therefore, glucosamine accumulation might be a cause of DPN independent of AR and the polyol pathway. Supporting this hypothesis, glucosamine injection into normal mice induced neurological abnormalities resembling DPN, including reduced sensory and motor nerve conduction velocities, decreased intraepidermal nerve fiber density, diminished Na^+^-K^+^-ATPase activity, and lower ATP levels in sciatic nerves. Furthermore, exogenously applied glucosamine accelerated cell death in a concentration-dependent manner (1 mM < 2.5 mM < 5 mM < 10 mM) and impaired insulin signaling (downregulating the expression of phosphorylated AKT and S6 ribosomal protein) and ATP production in IMS32 cells under both normoglycemic (5.6 mM) and hyperglycemic (30 mM) conditions (49). While the mechanism of glucosamine elevation in the PNS under diabetic conditions remains unclear, it is possible that glucosamine contributes to the development of DPN by inhibiting insulin signaling and ATP production in Schwann cells. Glucosamine can be metabolized to glucosamine-6-phosphate, a key intermediate in the hexosamine biosynthetic pathway, which is the second collateral glycolysis pathway (50, 51). Although there is currently no direct evidence of the glucosamine-induced activation of the hexosamine pathway in Schwann cells or its role in the pathogenesis of DPN (**Figure 2**), our ongoing study using AR-deficient Schwann cells (IKARS1) may help clarify these issues (Yako et al., in preparation).

### 2.3 Lipotoxicity

In addition to hyperglycemia, dyslipidemia resulting from obesity and type 2 diabetes plays a pivotal role in the development and progression of DPN (52). Several studies have identified Schwann cell lipotoxicity as a pathogenic factor in DPN (53, 54). Supporting these findings, palmitate (PA), a representative saturated fatty acid, induces cell death with caspase-3 activation in IMS32 cells in a dose-dependent manner (55). PA also upregulates the protein level of CCAAT/enhancer-binding protein homologous protein (CHOP), a common marker of endoplasmic reticulum (ER) stress, in both IMS32 cells and primary cultured Schwann cells (55), as well as in rat Schwann cell line RSC96 (56, 57). These findings suggest that PA induces apoptosis via ER stress in Schwann cells. Additionally, PA is recognized as a potent ligand for Toll-like receptor 4 (TLR4), a key activator of the innate immune response (58). The TLR4 signaling pathway is suggested to contribute to PA-induced cytotoxicity associated with ER stress (59) or inflammatory responses (60). However, a previous study (55) did not find the evidence of TLR4 involvement in PA-induced apoptosis in IMS32 cells. In contrast, oxidized low-density lipoprotein (oxLDL) under high-glucose conditions triggered the cell death and upregulated TLR4 mRNA and protein expression in IMS32 cells (61). Moreover, pretreatment with TAK-242, a selective TLR4 inhibitor, attenuated oxLDL-dependent cell death and the apoptotic caspase-3 pathway under high-glucose conditions. These findings suggest that hyperactivation of TLR4 signaling by oxLDL contributes to apoptotic cell death in IMS32 cells under hyperglycemic conditions. Given that elevated LDL levels are a risk factor for DPN (52, 62), TLR4 signaling represents a potential therapeutic target for DPN.

### 2.4 Oxidative stress

Reactive oxygen species (ROS), highly reactive forms of molecular oxygen, have detrimental effects on cells and tissues by inducing DNA fragmentation and the oxidation of proteins and lipids. Oxidative stress is defined as an imbalance between ROS production and accumulation, and the antioxidant defense system’s ability to detoxify ROS (63). Under diabetic conditions, ROS production in the PNS is enhanced by several metabolic disorders, including glucose autooxidation, polyol pathway hyperactivity, advanced glycation endproducts (AGEs)−receptor for AGEs (RAGE) interactions, and abnormal protein kinase C activity. Additionally, polyol pathway hyperactivity contributes to the loss of endogenous antioxidants, such as taurine and reduced glutathione (GSH). Osmotic pressure from sorbitol accumulation inhibits taurine intake, while AR competes with glutathione reductase (GR) for nicotinamide adenosine dinucleotide phosphate (NADPH); excessive NADPH consumption by AR can lead to GR inhibition and GSH depletion (25, 64). We (27) and others (65) have demonstrated enhanced AR activity/expression in IMS32 cells exposed to high-glucose conditions. In the latter study, increased AR activity was accompanied by elevated O_2_^-^ production, lipid peroxidation, and caspase 3 activity. These findings suggest causal relationships among the polyol pathway, oxidative stress, and apoptosis signaling.

Oxidative stress is a major therapeutic target for DPN, and α-lipoic acid (ALA) has been approved as an antioxidant treatment for DPN in several countries (66). In addition to ALA, omega-3 polyunsaturated fatty acids (ω-3 PUFAs), such as docosahexaenoic acid (DHA) and eicosapentaenoic acid (EPA), have demonstrated antioxidant and anti-inflammatory effects in diabetic conditions (67) and may be effective for DPN. Pretreatment with DHA and EPA alleviated IMS32 cell death caused by exposure to tert-butyl hydroperoxide (tBHP), an exogenous inducer of oxidative stress (68). The protective activities of DHA and EPA can be, at least in part, attributed to upregulation of endogenous antioxidant enzymes, such as heme oxygenase-1 and catalase. In another study, DHA protected PA-induced cell death in primary cultured rat Schwann cells through the activation of phosphatidyl inositol-3-kinase (PI3K)/AKT and mammalian target of rapamycin C2 kinase pathways (69); however, it remains unknown whether these pathways are involved in the DHA-induced upregulation of the antioxidant enzymes.

*Stachybotrys microspora* triprenyl phenols (SMTPs) are a family of triprenyl phenol metabolites derived from the fungus *S. microspore*. Among the SMTPs, SMTP-44D has been shown to exhibit antioxidant and anti-inflammatory effects on the nervous system (70). Administration of SMTP-44D ameliorated mechanical allodynia, thermal hyperalgesia, decreases in nerve conduction velocity and nerve blood flow, as well as increases in inflammatory molecules (e.g., tumor necrosis factor-α, interleukin (IL)-1β, IL-6, and malondialdehyde (MDA)) in the sciatic nerves of STZ-diabetic mice (71). In agreement with this study, SMTP-44D attenuated the upregulation of oxidative stress markers and inflammatory molecules, including NADPH oxidase-1, MDA, IL-6, and monocyte chemotactic protein 1, in IMS32 cells under hyperglycemic conditions (72). SMTP-44D also inhibited the enhanced activity of soluble epoxide hydrolase (sEH), which hydrolyzes epoxyeicosatrienoic acids (EETs) to dihydroxyeicosatrienoic acids (DHETs). Since EETs are potent endogenous signaling molecules associated with anti-inflammatory reactions, the protective effects of SMTP-44D in diabetic conditions may be mediated by its inhibition of sEH to sustain EET levels. These findings suggest the potential efficacy of SMTP-44D for DPN through its antioxidant and anti-inflammatory activities.

In addition to hyperglycemia, hypoglycemia due to intensive diabetes therapy and fluctuating glucose levels (glycemic variability) can trigger oxidative stress (73, 74). Recurrent short-term hyperglycemic and hypoglycemic conditions have been used as an *in vitro* model of glycemic variability, leading to enhanced oxidative stress and apoptosis in endothelial cells (75, 76), cardiomyocytes (77), and astrocytes (78), compared to constant hyperglycemic conditions. Similarly, intermittent short-term low and high glucose levels induced oxidative stress and apoptotic cell death in IMS32 cells (79). Since 4-phenyl butyric acid, an endoplasmic reticulum (ER) stress inhibitor, suppressed the cell death and oxidative stress induced by these conditions, glycemic variability-induced apoptosis and oxidative stress in Schwann cells may be mediated by ER stress responses (80). These findings suggest that good glycemic control to avoid hyperglycemia, hypoglycemia, and glucose fluctuation could prevent the onset and progression of DPN (81).

### 2.5 Pyruvate as a key molecule for ATP production under high glucose conditions

**Figure 3:**
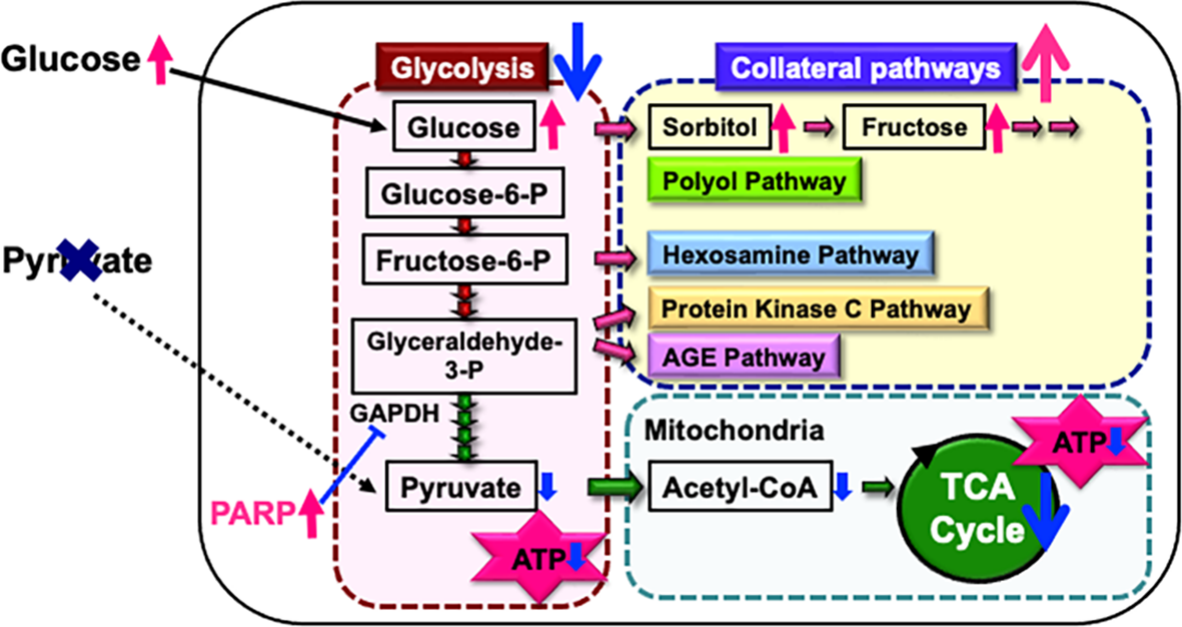
Possible mechanisms of IMS32 cell death under high-glucose and pyruvate-starved conditions. Deprivation of exogenous pyruvate reduces TCA cycle intermediates and mitochondrial ATP production, subsequently inhibiting glycolytic flux. Furthermore, enhanced activity of PARP under these conditions contributes to the suppression of glyceraldehyde-3-phosphate dehydrogenase (GAPDH) activity. This cascade results in the inhibition of glycolysis and a metabolic shift toward collateral pathways.

Endogenous pyruvate, produced from glucose through glycolysis, plays a key role in energy production, while exogenous pyruvate, taken up by cells via specific transporters, functions as a glycolysis accelerator and an antioxidant (82). However, the significance of pyruvate under diabetic conditions has remained unclear. In our study (83), exposure to high glucose (> 15 mM) in the absence of pyruvate led to rapid and extensive IMS32 cell death. Similarly, primary cultured adult rat DRG neurons, mouse mesangial MES13 cells, and human aortic endothelial cells underwent rapid cell death after exposure to the high-glucose conditions in the absence of pyruvate. Metabolome analysis revealed that the levels of pyruvate and certain TCA cycle intermediates, including 2-oxoglutarate, were significantly reduced in IMS32 cells under the high-glucose and pyruvate-starved conditions, and supplementation with these intermediates prevented cell death. Furthermore, exposure of IMS32 cells to these conditions resulted in a significant decrease in glycolytic flux and mitochondrial respiration, accompanied by enhanced flux through the polyol and other collateral glycolysis pathways. In our subsequent study using an inhibitor of poly-(ADP-ribose) polymerase (PARP), PARP activation under the high-glucose and pyruvate-starved conditions could contributes to the reduced glycolytic ATP production through the inhibition of glyceraldehyde-3-phosphate dehydrogenase (**Figure 3**). In contrast, PARP is unlikely to play a role in the impaired mitochondrial ATP production under those conditions (84). These findings suggest that exogenous pyruvate plays a crucial role in maintaining ATP production under high-glucose conditions through PARP-dependent glycolysis and PARP-independent TCA cycle in various cell types, including Schwann cells. Since supplementation of sodium pyruvate, an investigational drug for mitochondrial disease (85), ameliorated mechanical hypoalgesia and improved intraepidermal nerve fiber density in the lower limb of STZ-diabetic mice (Yako et al., in preparation), its potential therapeutic efficacy for DPN is promising.

## 3 IWARS1, IKARS1, and 1970C3 cells

IWARS1 and IKARS1 cells spontaneously arose from long-term primary cultures of adult wild-type and AR-deficient C57BL/6 mouse DRG and peripheral nerves, respectively (48). Schwann cell-enriched cultures were maintained under serum-free conditions in the presence of neuregulin-β, where fibroblasts ceased to grow. In the first attempt, IKARS1 cells were successfully established, but not IWASR1 cells. Therefore, 1970C3 cells, which were established from normal C57BL/6 mice, were used as a control for IKARS1 cells (18) until IWARS1cells were obtained in a second attempt. All the cell lines display distinct Schwann cell phenotypes, such as spindle-shaped morphology with intense immunoreactivity for glial cell markers and the synthesis and secretion of neurotrophic factors (18, 25). However, no studies have been conducted to determine whether these cells possess the capability to myelinate neurites in co-culture with neurons.

### 3.1 Physiological roles of AR

Enhanced AR activity and polyol pathway flux under hyperglycemic conditions have been implicated as a major cause of DPN and other diabetic complications. However, the physiological roles of the polyol pathway remain largely unclear. A recent study (86) suggests that the polyol pathway monitors intracellular glucose levels and regulates metabolic activities in response to glucose availability, but this function has not been verified in Schwann cells or the PNS. AR is a member of aldo-keto reductase (AKR) superfamily and participates in the detoxification of numerous aldehydic substances (87). Exposure to reactive aldehydes, including 3-deoxyglucosone, methylglyoxal (MG), and 4-hydroxynonenal (4HNE), significantly upregulated the mRNA expression of AKR1B7 and AKR1B8 in IKARS1 cells, but not in 1970C3 cells (18). Since no significant differences in viability between these two cells were observed after exposure to these aldehydes, aldehyde detoxification might be carried out by AKR1B7 and AKR1B8 in the absence of AR (*aka* AKR1B3). In addition to AKRs, the glyoxalase system is responsible for MG detoxification. Schwann cells deficient in glyoxalase 1 (GLO1) created using the CRISPR/Cas9 technique, did not show elevated MG concentrations. However, AR inhibition in GLO1-deficient Schwann cells increased intracellular MG levels and oxidative stress, suggesting that AR can compensate for the loss of GLO1 (88).

### 3.2 Glucoselysine as a novel AGE in the polyol pathway

In the second step of the polyol pathway, sorbitol is converted into fructose by sorbitol dehydrogenase (SDH). Fructose is further metabolized into dicarbonyl compounds such as 3-deoxyglucosone (3-DG) and MG, both recognized as potent glycating agents that contribute to the formation of AGEs. In addition to AGE-induced Schwann cell injury (89), 3-DG and MG have been shown to exert direct toxicity against Schwann cells (38, 90, 91). Recent studies have highlighted the AGEs produced from both exogenous (diet-derived) and endogenous (polyol pathway-derived) fructose as novel pathogenic factors in various diseases, including diabetic complications (92). Glucoselysine (GL) has been identified as a novel AGE primarily produced from fructose, and GL levels were found to increase in the eye lenses of STZ-diabetic rats in a time-dependent manner (93). Furthermore, exposure to high-glucose conditions increased both intracellular and extracellular GL levels in IWARS1 cells, but not in IKARS1 cells (28). Since the polyol pathway is absent in IKARS1 cells, it is likely that GL is produced via the polyol pathway and released from Schwann cells under diabetic conditions. A clinical investigation involving patients with type 2 diabetes and healthy participants revealed that serum GL levels were significantly higher in the diabetic patients. Moreover, GL levels in these patients were correlated with the duration of diabetes, as well as the presence of renal dysfunction and vascular complications (28). GL is expected to be a valuable biomarker for assessing the severity of DPN and other complications, as well as a potential therapeutic target in the polyol pathway-related pathogenesis.

### 3.3 Proteostatic disturbances

In addition to combined use with IKARS1 cells, IWARS1 cells can also be used individually as one of the mouse Schwann cell lines, similar to IMS32 cells. In our recent study (26), findings from a *Drosophila* model of DPN were further validated through proteome analyses using IWARS1 cells. High-sugar diet (HSD)-fed flies developed hyperglycemia and reduced insulin sensitivity, subsequently displaying DPN-like phenotypes, such as impaired noxious heat avoidance and atrophy of leg sensory neurons. Genetic screening of these flies identified the proteasome 26S subunit, non-ATPase 9 (PSMD9), as one of the modifier genes associated with impaired heat avoidance. PSMD9 gene polymorphisms have been linked to DPN risk (94), and PSMD9 is involved in proteasome activity (95). These findings suggest that proteasome activity via PSMD9 is linked to sensory dysfunction in HSD-fed flies. Supporting this hypothesis, glia-specific PSMD9 knockdown or proteasome inhibition suppressed the effects of HSD. Additionally, treatment with Ixazomib, an oral proteasome inhibitor, alleviated heat avoidance impairment and prevented atrophic changes in leg sensory neurons in HSD-fed flies. Subsequent proteome analyses using IWARS1 cells revealed that Ixasomib upregulated heat shock proteins (HSPs), including HSP40 and HSP70, suggesting that HSPs play a role in Ixazomib’s restorative effects. Furthermore, glia-specific knockdown of HSP40 or related genes negated the effects of Ixazomib. The glial proteasome is thus a promising therapeutic targets for DPN, and the exosomal secretion of HSPs from the glia may mediate the protective effect of proteasome inhibition (96).

## 4 IFRS1 cells

IFRS1 cells spontaneously arose from long-term primary cultures of adult Fischer 344 rat DRG and peripheral nerves under serum-free conditions in the presence of neuregulin-β and forskolin (17). IFRS1 cells display distinct Schwann cell phenotypes, including a spindle-shaped morphology with intense immunoreactivity for glial cell markers, as well as the synthesis and secretion of neurotrophic molecules (97, 98). Additionally, IFRS1 cells have been shown to myelinate neurites in co-culture with primary cultured adult rat DRG neurons (17), NGF-primed PC12 cells (21), rat neural stem cell-derived neurons, mouse embryonic stem cell-derived motor neurons (99), and NSC-34 motor neuron-like cells (100). Due to their capability to form myelin structure, IFRS1 cells are advantageous for studying the molecular mechanisms of myelination (101, 102) and demyelination (103, 104).

Unlike IMS32 cells, IFRS1 cells do not appear useful for studying the polyol pathway; exposure of IFRS1 cells to high-glucose conditions did not increase intracellular sorbitol and fructose levels (Sango et al., unpublished data). However, these cells have been utilized to explore other pathogenic factors in DPN, including impaired insulin signaling, glycation, and oxidative stress-induced autophagy.

### 4.1 Insulin signaling

Insulin plays a central role in regulating blood glucose levels, and impaired insulin signaling in muscle and adipose tissue is a known contributor to type 2 diabetes. It is important to note that insulin receptors are present not only in these tissues, where insulin regulates blood glucose uptake, but also in neurons and Schwann cells in the PNS (105, 106), where blood uptake occurs in an insulin-independent manner. These findings suggest that insulin may have neurotrophic and neuroprotective roles (107, 108), and that impaired insulin signaling in the PNS may contribute to the pathogenesis of DPN, as discussed in the context of glucosamine toxicity [2.2].

Insulin receptors have been identified in IFRS1 cells, where insulin application induces phosphorylation of AKT, mitogen-activated protein kinase kinase (MEK), and extracellular signal-regulated kinase (ERK) (22). Further studies using specific inhibitors for PI3K/AKT and MEK/ERK signaling pathways revealed that short-term insulin treatment promotes IFRS cell proliferation, likely through the PI3K/Akt pathway rather than the MEK/ERK pathway. In contrast, long-term insulin exposure upregulated the protein expression of MPZ via the MEK/ERK pathway and myelin basic protein (MBP) via the PI3K/AKT pathway. These findings indicate that the PI3K/AKT and MEK/ERK pathways are involved in insulin-induced proliferation, survival and differentiation of Schwann cells in distinct ways. Supporting this idea, MEK/ERK signaling has been shown to regulate Schwann cell mitosis (109), while disrupting insulin-PI3K/AKT signaling in Schwann cells leads to sensory neuropathy with impaired myelination (110). Targeting PI3K/AKT and MEK/ERK pathways in the PNS may thus represent a novel therapeutic strategy against DPN (111).

### 4.2 Glycation

Galectin-3 (GAL-3), a member of the β-galactoside-binding animal lectin family, is involved in various cell-to-cell and cell-to-matrix interactions. Recent studies have highlighted both physiological and pathological roles of GAL-3 in nervous tissue (112). Similar to RAGE, GAL-3 is recognized as an AGE-binding protein; however, the actions of these two proteins appear to be oppositional under diabetic conditions. While the AGEs-RAGE interaction can contribute to the development of DPN and other diabetic complications (113, 114), GAL-3 may act as a cytoprotective molecule by alleviating AGEs toxicity (115, 116). Exposure of IFRS1 cells to high glucose (30 mM) and 3-deoxyglucosone (3-DG; 0.2 mM), a precursor of AGEs, induced an upregulation of GAL-3 expression. Additionally, treatment with exogenous recombinant mouse GAL-3 (1 µg/mL) led to an upregulation of the anti-apoptotic marker Bcl-2 and a downregulation of the oxidative stress marker 4HNE in IFRS1 cells (91). These findings suggest that increased GAL-3 expression in Schwann cells under diabetes-mimicking conditions may play a pivotal role against DPN progression, although its precise mechanisms of action remain to be elucidated.

In addition to AGEs, their precursors−including MG, glyoxal, 3-DG, glyceraldehyde and glycolaldehyde (GA)−have shown detrimental effects on neurons and Schwann cells (38, 91, 117, 118). Among these, GA has been identified as the most harmful metabolite for IFRS1 cells and ND7/23 sensory neuron-like cells (118). Further analysis suggests that c-jun N-terminal kinase (JNK) and p-38 mitogen-activated kinase (p-38 MAPK) signaling pathways are involved in GA-induced ND7/23 cell death. It remains unclear whether GA toxicity against IFRS1 cells (118) and primary cultured rat Schwann cells (117) is associated with the activation of JNK and/or p-38 MAPK pathways. Nevertheless, GA and GA-induced intracellular AGEs accumulation may lead to ER stress, thereby being potentially contributing to DPN and other complications (117, 119).

### 4.3 Oxidative stress-induced autophagy

Autophagy is a catabolic process that maintains cellular homeostasis by eliminating damaged intracellular components through lysosomal degradation. Dysregulation of autophagy contributes to the progression of various diabetic complications, including DPN (120). It has been suggested that either excessive or impaired autophagy in Schwann cells under hyperglycemic conditions is associated with DPN pathogenesis (11, 121). Since autophagy is activated in response to increased ROS production (122), ROS accumulation induced by hyperglycemia in Schwann cells may enhance autophagic reactions.

The antioxidant activities of DHA toward IMS32 cells (68) and its potential efficacy in DPN has been described in the context of oxidative stress [2.4]. In a subsequent study (123), DHA pretreatment was shown to alleviate tBHP-induced oxidative stress, excessive autophagy, and cell death through the AMP-activated protein kinase-dependent signaling pathway in IFRS1 cells. In another study, melatonin reduced high glucose-induced ER stress and autophagy in RT4-D6P2T rat Schwann cells (124). These findings suggest that excessive autophagy induced by oxidative and/or ER stress in Schwann cells under diabetic conditions may be a viable therapeutic target for DPN. However, further evidence from animal and clinical studies is required to verify this hypothesis.

## 5 Translational findings in patients with DPN

This article summarizes the major findings on both classical and novel pathogenic factors of DPN identified using rodent Schwan cell lines established in our laboratory, including IMS32, 1970C3, IWARS1, IKARS1, and IFRS1. Additionally, we introduce potential therapeutic approaches targeting these factors. Some of these findings may have translational relevance for DPN in humans.

### 5.1 Schwann cell differentiation

Schwann cell dedifferentiation is considered a key pathogenic factor in peripheral nerve disorders, including DPN. Conversely, promoting Schwann cell differentiation may have therapeutic potential for DPN [2.1]. A promising candidate in this regard is ONO-2910 ((E)-3-(2-((5-(3-(phenylsulfonamido)phenyl)pent-4-en-1-yl)oxy)phenyl)propanoic acid, a novel Schwann cell differentiation enhancer developed by Ono Pharmaceutical Co., LTD, Osaka, Japan. Clinical trials for ONO-2910 in patients with DPN are currently underway.

### 5.2 Imeglimin as a promising antioxidant remedy for DPN

Imeglimin, a novel anti-hyperglycemic agent available in Japan, exerts a unique mechanism targeting mitochondrial dysfunction. Mitochondrial dysfunction is a key contributor to impaired glucose uptake in muscles, excessive gluconeogenesis in the liver, and increased pancreatic β-cell apoptosis. By protecting mitochondrial function and reducing ROS production, imeglimin improves glycemic control (125). Given that oxidative stress is a significant pathogenic factor for DPN and other diabetic complications [2.4], imeglimin may offer benefits beyond glycemic control, ameliorating these conditions. Our ongoing study demonstrates that imeglimin alleviates oxidative stress and apoptotic cell death in IMS32 cells exposed to high-glucose, low-glucose, and recurrent glucose fluctuation conditions (Kato et al., in preparation). Furthermore, a recent clinical study highlighted imeglimin’s favorable effects on body weight and lipoprotein profiles in type 2 diabetes patients (126). However, further studies are needed to evaluate its efficacy for chronic complications, including DPN.

### 5.3 The efficacy of GLP-1 receptor agonists for DPN

Exendin-4 (Ex-4), a glucagon-like peptide-1 receptor agonist (GLP-1RA), has demonstrated efficacy in ameliorating DPN in STZ-diabetic mice, independent of its blood glucose-lowering effects (127). Consistently, Ex-4 has been shown to enhance the survival and neurite outgrowth of rat DRG neurons (128), promote survival/proliferation and migration of IFRS1 Schwann cells, and myelination in DRG neurons-IFRS1 co-cultures (101). These findings suggest neuroprotective properties of Ex-4 and its potential role in targeting DPN. However, evidence supporting the efficacy of Ex-4 and other GLP-1RAs in humans remains controversial (129). A recent study associated Ex-4 therapy with improvements of nerve excitability in patients with type 2 diabetes (130). Additionally, GLP-1RA therapy has shown improvements in nerve conduction velocities and axonal excitability, and morphological abnormalities assessed using peripheral nerve ultrasonography in DPN patients (131). Nonetheless, further clinical research is required to establish the therapeutic efficacy of GLP-1RA for DPN.

## 6 Conclusion

**Figure 4:**
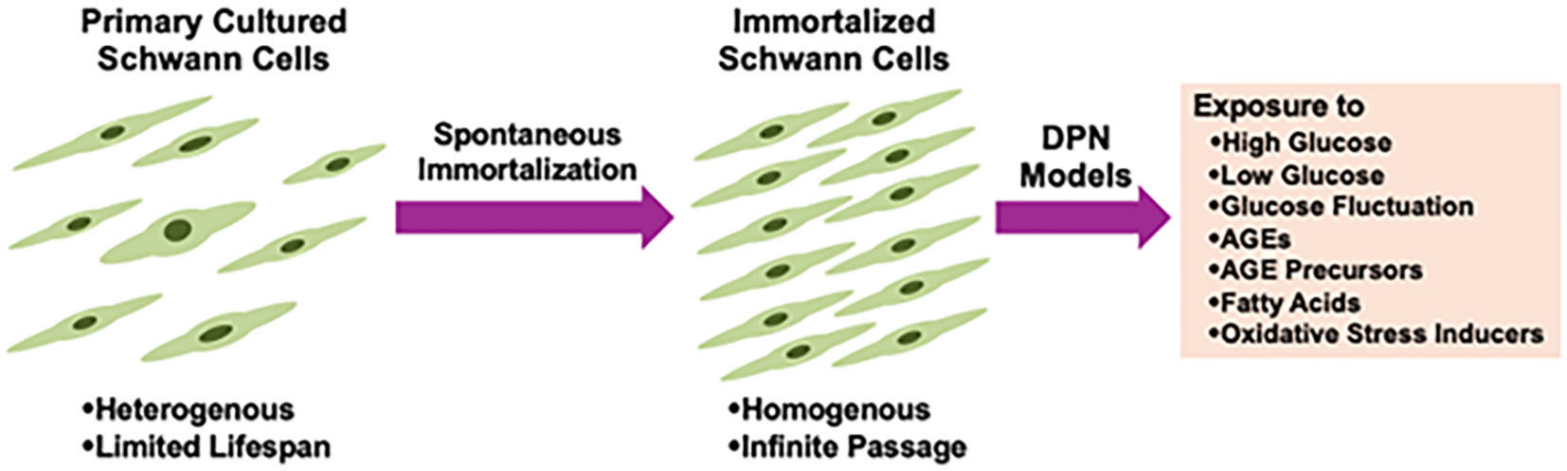
Immortalized Schwann cells as useful tools for the study of DPN.

Despite extensive research efforts, no FDA-approved disease-modifying therapies for DPN currently exist. We hope this article will aid researchers studying DPN in gaining a deeper understanding of the unique characteristics of the immortalized Schwann cells described here and facilitate their use in developing effective treatments (**Figure 4**).
